# The Past, Present, and Future in Antinuclear Antibodies (ANA)

**DOI:** 10.3390/diagnostics12030647

**Published:** 2022-03-07

**Authors:** Juan Irure-Ventura, Marcos López-Hoyos

**Affiliations:** 1 Immunology Department, University Hospital Marqués de Valdecilla, 39008 Santander, Spain; juan.irure@scsalud.es; 2 Autoimmunity and Transplantation Research Group, Research Institute “Marqués de Valdecilla” (IDIVAL), 39011 Santander, Spain; 3 Molecular Biology Department, University of Cantabria, 39011 Santander, Spain

**Keywords:** antinuclear antibodies (ANAs), systemic autoimmune rheumatic diseases (SARDs), indirect immunofluorescence (IIF), solid-phase assays, particle-based multi-analyte technology (PMAT), machine learning

## Abstract

Autoantibodies are a hallmark of autoimmunity and, specifically, antinuclear antibodies (ANAs) are the most relevant autoantibodies present in systemic autoimmune rheumatic diseases (SARDs). Over the years, different methods from LE cell to HEp-2 indirect immunofluorescence (IIF), solid-phase assays (SPAs), and finally multianalyte technologies have been developed to study ANA-associated SARDs. All of them provide complementary information that is important to provide the most clinically valuable information. The identification of new biomarkers together with multianalyte platforms will help close the so-called “seronegative gap” and to correctly classify and diagnose patients with SARDs. Finally, artificial intelligence and machine learning is an area still to be exploited but in a next future will help to extract patterns within patient data, and exploit these patterns to predict patient outcomes for improved clinical management.

## 1. Introduction

Autoantibodies are a hallmark of autoimmunity and, specifically, antinuclear antibodies (ANAs) together with anti-dsDNA antibodies and extractable nuclear antigens (ENAs) are the most relevant autoantibodies present in systemic autoimmune rheumatic diseases (SARDs), since they can be relevant for the classification, diagnosis, and monitoring of patients with connective tissue diseases (CTDs). Therefore, in the present work a review of the past, present, and future of ANAs was performed, showing the evolution that has taken place from aspects related to the services involved in ANA requests to the different techniques that have been developed for their determination.

## 2. Past of ANA Testing

The first evidence of the existence of ANAs was the description of the LE-cell phenomenon by Hargraves et al. in 1948 [1]. Together with the classic LE-cell phenomenon, which is based on the phagocytosis of intact nuclei by mature polymorphonuclear leukocytes in the bone marrow, the examination of the bone marrow from systemic lupus erythematosus (SLE) patients revealed the adherence of nuclei to and phagocytosis by immature myeloid cells. Following the description of LE cells, the identification of anti-DNA antibodies in 1957 [2,3] took place. The discovery of anti-Sm antibodies by Kunkel and Tan in 1966 provided more evidence that autoantibodies present in SLE patients recognize nuclear structures differently from nucleosomes [4]. Considering that the presence of autoantibodies in serum samples could have diagnostic value, different techniques such as indirect immunofluorescence (IIF), immunodiffusion, hemagglutination, and complement fixation were developed in the following 20 years. In addition, in the 1970s counter-immunoelectrophoresis, Western blot, and enzyme-linked immunosorbent assays (ELISA) were also developed [5]. Firstly, IIF was described by Coons, Kaplan, and Weller in the early 1950s [6], with cryopreserved sections of rodent tissues being the main substrate used for many years [2,7,8,9]. It was not until the mid-1970s when it was discovered that human tissue culture cells, such as human epithelial type-2 cells (HEp-2 cells), derived from laryngeal carcinoma, were better than primary organ sections, since the production of this type of cell in large number was easier and they had bigger nuclei and expressed antigens in various stages of the cell cycle [10,11]. Therefore, the mid-1970s could be considered the beginning of the ANA-detection explosion using as substrate HEp-2 cells in SARDs.

## 3. Present of ANA Testing

### 3.1. Indirect Immunofluorescence (IIF) Assays Using HEp-2 Cells

**Figure 1:**
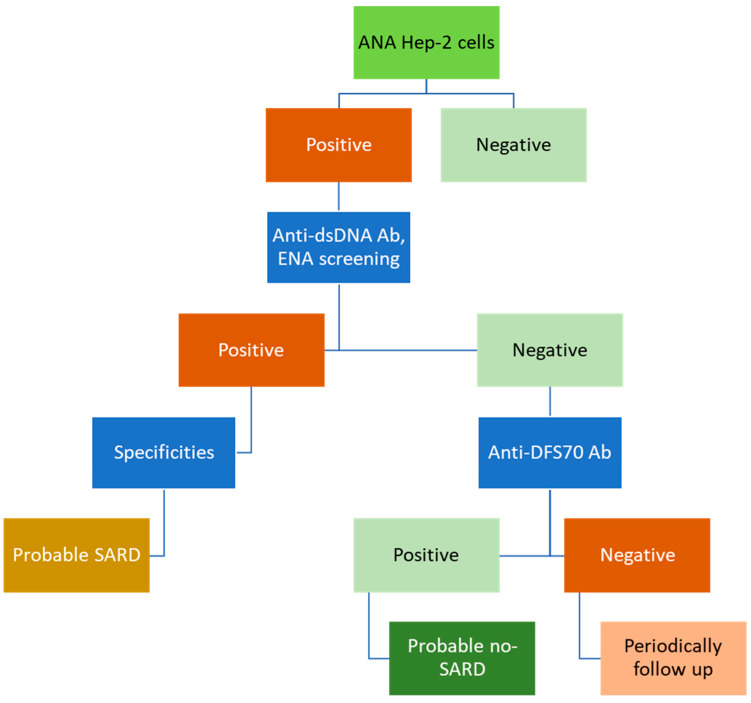
Conventional algorithm for ANA screening at present by using screening with IIF on HEp-2 cells.

IIF assays using HEp-2 cells is the gold standard approach to performing screening for ANAs in SARDs. Figure 1 shows a conventional algorithm that could be used for this purpose. When a positive ANA result is obtained on HEp-2 cells, further studies are necessary to identify the specific autoantibodies responsible of the IIF pattern, such as anti-dsDNA or anti-ENA antibodies. While a positive result on these second-step assays could point towards a possible SARD, a negative one in a patient with ANAs on HEp-2 cells might require looking for the presence of anti-dense fine speckled 70 (DSF70) antibodies in order to discard the existence of SARDs or to follow up periodically. A relevant aspect is that this screening for ANAs should be accompanied by clinical information on the patients.

Considering these aspects, new questions regarding ANA testing have been raised. Should we continue performing diagnoses based on classic algorithms despite being in the era of new autoimmune diagnostic platforms? Trying to answer this question, we need to know how ANA requests have evolved over the years.

Historically, in 1950s only rheumatologists and clinical immunologists ordered ANA tests for diagnosis of SARDs. Over the years, nephrologists, internists, and gastroenterologists joined in using ANA requests and currently, almost any clinician, and even general practitioners request the determination of ANAs [12].

As a consequence of the increase in the number of requesting services, in the 1950s the pretest probability for ANA detection was very high and over the years it has been significantly reduced, reaching a very low pretest probability today [13]. As is known, the posttest probability for disease associated with a particular test result can be estimated based on the pretest probability and the likelihood ratio for that particular test result [14,15]. Therefore, due to the low pretest probability that currently exists, the posttest probability for ANAs is also low.

The increase in the number of requesting services for ANA assays has been due to better knowledge of the pathophysiology of the different diseases, where the involvement of the autoimmune processes is better understood. In the middle of the 20th century SLE was practically the only autoimmune disease where the importance of ANAs was known. Over the years, it has been observed that there are other diseases, such as, Sjögren syndrome, mixed connective tissue disease, rheumatoid arthritis, antiphospholipid syndrome, systemic sclerosis (SSc), inflammatory myopathies (IIM), glomerulonephritis, vasculitis, encephalitis, autoimmune hepatitis, primary biliary cholangitis, inflammatory bowel disease, and interstitial lung disease, where autoimmune phenomena and ANAs also play a very important role [16,17,18,19].

**Figure 2:**
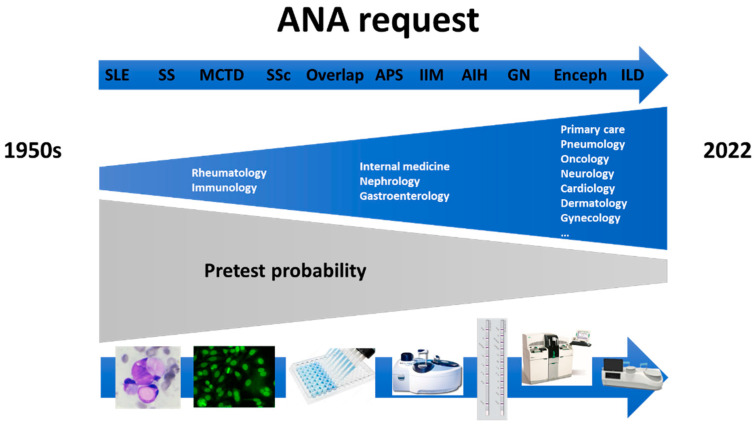
Evolution of ANA request from the 1950s to 2022 according to the involvement of autoimmune phenomena in different diseases and technological development.

This increase in knowledge of the different autoimmune diseases has occurred thanks to the great technological development, from LE cells, through IIF, ELISA, chemiluminescence or immunoblot assays, to multi-analyte technologies (Figure 2).

Taking all this into account, and as shown by the conventional algorithm for ANA screening, the first step in the study of ANAs is IIF using HEp-2 cells (Figure 1). The position statement of the American College of Rheumatology (ACR), published in 2010 and countersigned in 2019 (also by European League Against Rheumatism, EULAR), according to the methodology of testing for ANAs shows that the ACR and EULAR support the ANA test using HEp-2 cell substrate, as the gold standard for ANA testing. In addition, this statement mentions that hospital and commercial laboratories using alternative bead-based multiplex platforms or other solid-phase assays for detecting ANAs must provide data to ordering healthcare providers on request that the alternative assay has the same or improved sensitivity compared to IIF assay. Laboratories should specify the methods used for detecting ANAs when reporting their results [20].

Despite the position of the ACR, and based on different publications, different questions of whether we should rethink this position are arising. Is the IIF still the gold standard or should be replaced by solid-phase assays? Is it time to compare solid-phase assays and automated IIF for detection of ANAs? [21,22,23]. Can solid-phase assays replace immunofluorescence for ANA screening? [24].

However, despite the great development of alternative techniques for the study of ANAs, the 2019 EULAR/ACR classification criteria for SLE maintain the presence of ANAs of HEp-2 cells as an entry criterion for SLE patients [25,26]. Therefore, the time has not yet come to unseat the IIF as the gold standard assay for ANA detection.

IIF assay using HEp-2 cells is laborious process, that needs skilled operators. Despite its sensitivity, HEp-2 IIF has a high inter-observer variability and low specificity [12], due to the fact that ANAs can be present in patients with SARDs, but also in non-rheumatic diseases and in healthy individuals [27,28].

A positive result for ANAs in HEp-2 cells should always be communicated to clinicians in a standardized way, including endpoint titer and immunofluorescence pattern, together with anti-cell (AC) nomenclature, and remarking reflex tests and possible autoantibody associations, adding value to laboratory findings and helping with clinical decisions [29,30].

The International Consensus on ANA Patterns (ICAP) working group have made efforts to harmonize the HEp-2 pattern nomenclature, describing 29 different HEp-2 cell patterns, grouped into nuclear, cytoplasmic, and mitotic and also distinguishing between competent and expert-level patterns [31,32]. Due to ANA testing by HEp-2 IIF revealing reactivity not only to nuclear antigens but also to cytoplasmic and mitotic antigens, the term anti-cell antibodies, proposed by ICAP, is being increasingly used by researchers [31]. However, this term is only slowly being implemented in clinical practice. The main ANA staining patterns informed by most of the hospitals include nuclear patterns, such as homogeneous (AC-1), speckled (AC-4, 5), nucleolar (AC-8-10), and centromere (AC-3), together with cytoplasmic reticular/AMA (mitochondria-like) (AC-21). However, the remaining patterns, whether nuclear, cytoplasmic, or mitotic, sometimes referred to as rare or infrequent immunofluorescence patterns, should not be forgotten. These rare immunofluorescence patterns of autoantibodies on HEp-2 cells defined by ICAP help to identify different autoimmune diseases, including organ-specific and non-organ-specific diseases, in the absence of associated specificities [33]. Likewise, it should be taken into account that we are facing an explosion of autoantibodies [34], with more than 200 in SLE [35,36], 60 in SSc [37,38,39], and 25 in IIM [40,41,42].

Taking all of this into account, an important consideration regarding ANAs is the fact that whenever a positive result is obtained, both the IIF pattern and the titer must be reported, including nuclear, cytoplasmic, and mitotic patterns. As is known, estimates of the title-specific likelihood ratios of HEp-2 IIF suggest that there is a positive correlation between the likelihood ratio, and therefore, the positive predictive value, and the antibody titer. This is demonstrated by the fact that the clinical and diagnostic value of a positive ANA HEp-2 IIF result at 1:80 titer (likelihood ratio of 0.5) is different to that of a titer of 1:640 (likelihood ratio of 19). In other words, the probability that a patient with SLE will have a positive result for ANAs on HEp-2 cells by IIF at a titer of 1:640 is 19 times higher than a healthy subject. In the same way, the probability that ANAs on HEp-2 cells will be detected by IIF at a titer of 1:80 on samples from a healthy subject is twice that of samples from patients with SLE [43,44].

The international recommendations for the assessment of ANAs defines the HEp-2 IIF cut-off value as the titer that corresponds to the 95th percentile of local age-matched and gender-matched healthy individuals [45] which is in agreement with a titer of 1:160 in an international study carried on healthy subjects [27]. However, the same experts from the international group who developed these recommendations recognized that the sensitivity for the diagnosis of SARDs associated with the presence of ANAs on HEp-2 cells by IIF at a titer of 1:160 is moderate, and therefore obtaining a negative result using the mentioned dilution does not necessarily rule out the presence of disease [45]. If we focus on the EULAR/ACR 2019 classification criteria for SLE we can observe that the presence of ANAs has been included as an entry criterion for SLE classification. Specifically, the positivity for ANAs on HEp-2 cells has been stablished in a titer of 1:80 [46]. The steering committee for the EULAR/ACR 2019 classification criteria for SLE concluded, based on systematic review and meta-regression analysis of the performance of HEp-2 IIF antibody testing for classifying SLE, that the sensitivity of HEp-2 IIF for ANA detection using a titer of 1:80 is high enough (97.8%) to be considered as an entry criterion [25,26,47]. However, it should be noted that the specificity associated with the 1:80 cut-off titer is low (74.7%) [47]. Using the mentioned dilution, up to 13.3% of heathy individuals show ANA-positive results [27]. The high sensitivity obtained using a cut-off point of 1:80 is the key aspect that allows the recommendation of the presence of ANAs on HEp-2 cells by IIF as an entry criterion for the classification of SLE patients. Nevertheless, as mentioned above, due to the low likelihood ratio, and therefore low PPV, a positive result obtained at a titer of 1:80 should be interpreted with caution when it is used for diagnostic purposes In this type of patient, where a low ANA result is obtained using HEp-2 cells, the presence of clinical manifestations should have a greater importance in the diagnostic workflow in order to avoid misdiagnosis and the use of treatments that are unnecessary [44].

Another important aspect is the relevance of a negative ANA result obtained using HEp-2 cells. It must be taken into account that HEp-2 cells might miss the presence of certain antibodies, such as antibodies to SSA/Ro60, Ro52 (also known as TRIM21), ribosomal P, Jo1, and other amino-acyl tRNA synthetases, SRP, or MDA5, among others, due to the low abundance of the antigen or because of the fixation method [41,43,48,49,50,51]. Therefore, if the presence of a SARDs associated with some of these autoantibodies is suspected, a negative result on HEp-2 IIF assay should be confirmed using alternative diagnostic assays, such as solid phase assay (SPA).

It is also important to highlight the complexity of ANAs in clinical practice, in terms that ANAs are obviously present in SARDs, but also in many other conditions, such as infectious diseases [52,53], inflammatory processes [54], and also the presence of ANAs increase with the age [55,56]. As the age of the patients increases, the number of associated diseases also increases and with it the request for ANAs [57]. However, the age of onset of autoimmune diseases does not usually occur at advanced ages, but at younger ages [58,59].

### 3.2. Solid-Phase Assays (SPAs)

IIF assay used for ANA detection is a screening method. Therefore, whenever there is a positive result or a negative result that is accompanied by symptoms suggestive of SARDs, SPAs must be carried out to determine the specificity of the autoantibodies.

**Figure 3:**
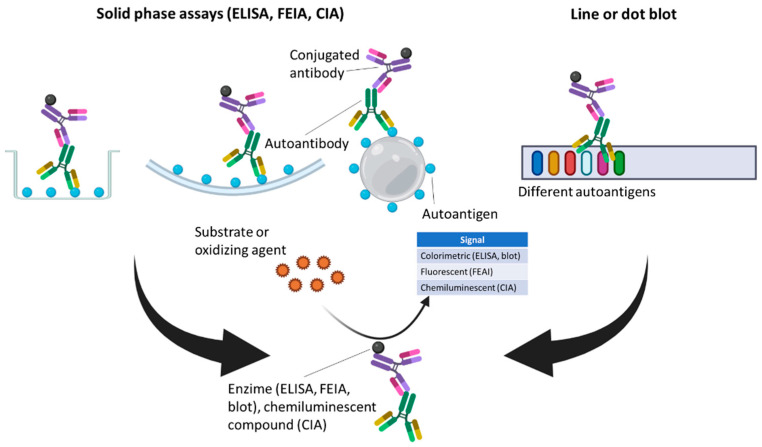
Solid-phase assays (ELISA, FEIA, and CIA) and line or dot blots as screening and confirmation methods for autoantibody detection.

Over recent years, different SPAs, including ELISA, fluorometric enzyme-linked immunoassay (FEIA), and chemiluminescence immunoassay (CIA), have been increasingly introduced in clinical laboratories to screen for ANA-associated SARDs, and also to confirm the specificity of autoantibodies in cases of positive screening results. Moreover, line blots and dot blots should be considered but they are typically not used to screen for ANA-associated SARDs and are instead usually used for the identification of specific autoantibodies [60]. In these SPAs, when screening assays are performed, the solid phase, a plate in the case of ELISA and FEIA, or a bead in the case of CIA, is coated with a mixture of relevant, purified or recombinant, specific autoantigens towards autoantibodies can be present in serum samples. If, However, confirmation assays are being carried out, the solid phase is coated with a single specific autoantigen. If autoantibodies are present in a patient serum, they attach to their corresponding autoantigen and are bound by a detection antibody linked to an enzyme that generates a colorimetric reaction in the case of ELISA, a fluorescent reaction in the case of FEIA, or the detection antibody can be linked to a chemiluminescent compound in the case of CIA. In the case of dot or line blots, a nitrocellulose membrane is coated with different autoantigens toward autoantibodies which can be detected using a detection antibody linked to an enzyme that generates a colorimetric reaction, as in the case of ELISA (Figure 3).

In comparison with IIF assay, SPAs are less sensitive but more specific for the screening of ANA-associated SARDs [43,61]. However, it is essential to take into account how the different cut-off points of the assays are chosen in order to consider a positive result, since the differences observed in the performance between assays are largely related to these. Thus, in a meta-analysis of paired studies and considering the cut-off value for HEp-2 IIF as 1:80 the sensitivity of IIF, FEIA, and CIA was 89.2%, 78.5%, and 85.9%, respectively, and the specificity was 70.9%, 93.6%, and 86.1%, respectively, for ANA-associated SARDs [22]. In contrast, studies that consider the cut-off point as the value that corresponds to a specificity of 95%, showed that HEp-2 IIF, FEIA, and CIA for ANA-associated SARDs presented similar sensitivities of 79%, 82%, and 78%, respectively [13]. Regarding ELISA for ANA-associated SARDs, is also important to know the type of ELISA used, as well as the antigenic source, since differences in the sensitivity and specificity are present. In this way, ELISA based on a mixture of purified antigens showed lower sensitivity (76%) but higher specificity (90.4%) than HEp-2 IIF assay (87.4% of sensitivity and 72.3% of specificity). However, ELISA that have HEp-2 extract as antigenic source had higher sensitivity (90%) but lower specificity (50%) than HEp-2 IIF assay [62]. Therefore, it is essential to know the technique we are using and to know how the cut-off points have been defined in order to correctly assess the results obtained.

Sensitivity and specificity values, in addition to the type of technique used, vary depending on the specific disease, since SPAs are more sensitive for Sjögren syndrome patients that HEp-2 IIF, but this technique is more sensitive than SPAs for SSc and SLE patients. Moreover, the sensitivity of SPAs and HEp-2 IIF for IIM is relatively low, independently of the assay used [13,61,62,63,64,65,66,67]. These differences could be due to HEp-2 cells having approximately 100–150 possible autoantigens, which can be considered the bigger array of autoantigens, while not all specific autoantigens are included in SPAs. Therefore, in addition to considering the type of technique used, the type of disease must be taken into account, since the results depends on it.

Providing results from HEp-2 IIF and SPAs should not be considered as independent, they should not be evaluated in an isolated manner, since they provide complementary information in the study of ANA-associated SARDs. Different studies that evaluate the diagnostic test accuracy of SPAs used alone or in combination with HEp-2 IIF for ANA-associated SARDs showed that a double-positive test result presented a higher likelihood ratio, and therefore a higher PPV, for ANA-associated SARDs, than a positive test result obtained from just one of the mentioned techniques. Specifically, Orme, M. E. et al. showed, in a systematic review, that comparing the use of FEIA alone or in combination with HEp-2 IIF the likelihood ratio for ANA-associated SARDs was higher when both assays were used. Specifically, the likelihood ratio was 26.2 for a double- positive result, 14.4 for positivity by FEIA alone, or 5.1 when the positivity was obtained using HEp-2 IIF alone. In the same way, the likelihood ratio was lower when a negative test result was obtained using both assays (0.15 for negativity by FEIA and HEp-2 IIF) than when only a single assay provided a negative test result (0.21 for negativity by HEp-2 IIF alone and 0.33 for negativity by FEIA alone) [67]. Another study performed by Claessens, J. et al. but comparing CIA and HEp-2 IIF found similar results. The likelihood ratio was higher when double positive results by HEp-2 IIF and CIA were obtained (12.2) in comparison with the positivity by CIA (7.3) or HEP-2 IIF (2.4) alone [13].

It is well known that in some cases the use of HEp-2 IIF is able to detect the presence of antibodies in samples from patients with SARDs associated with ANAs that are undetectable using SPAs. Therefore, the information provided by both types of assays (HEp-2 IIF and SPA) should not be used in isolation but in combination, since it has the advantage that either of the two types of assays may be able to detect certain antibodies that might not be detected by the other assay. Eight percent of patients with SARDs associated with the presence of ANAs have a negative test result by FEIA but a highly positive test result when HEp-2 IIF is used. Conversely, the likelihood ratio for SARDs associated with the presence of ANAs is higher in those patients with positive results near the cut-off value or a negative test result by HEp-2 IIF and a positive result by FEIA, than in patients who present the combination of a weakly positive or negative test result by HEp-2 IIF and a negative result by FEIA. Likewise, and as expected, the likelihood ratio for ANA-associated SARDs is highest in patients with positive results by HEp-2 IIF and FEIA assays [23]. 

Therefore, combining the results obtained using SPAs and HEp-2 IIF is more powerful than performing either assay alone, and could have a diagnostic value, since double-positive results or double-negative results by both techniques can better confirm or rule out disease, respectively.

## 4. Future of ANA Testing

**Figure 4:**
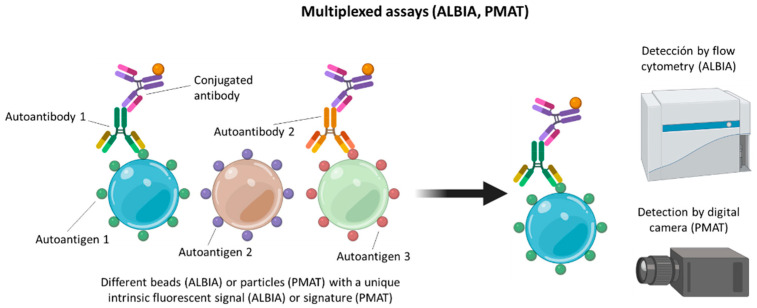
Multiplexed assays (ALBIA and PMAT) for autoantibody detection.

Without forgetting the mentioned SPAs, multiplexing SPAs are probably the future of the detection techniques for ANA testing in SARDs. Nevertheless, addressable laser bead immunoassay (ALBIA) and particle-based multianalyte technology (PMAT), which are the two main multianalyte techniques are already available [68,69]. In ALBIA, each autoantigen is coupled to a bead with a distinctive intrinsic fluorescent signal. This approach enables the simultaneous, multiplexed measurement of autoantibodies to various autoantigens using flow cytometry. In PMAT, each of the analytes is bound to paramagnetic particles that have a unique color code. Each unique color code allows the identification of analytes in the process, since once the samples have been processed, the paramagnetic particles are isolated and illuminated by the system using two LEDs. A red LED is used to identify the analyte and a green LED is used to determine the status of the analyte (positive or negative). Finally, a digital camera captures images of all analytes (Figure 4).

However, the most important aspect of the future of autoimmunity, and therefore of the future of ANA-associated SARDs, is the search for new, useful biomarkers that will help us to close the “seronegative gap” in order to better classify and diagnose patients, specifically in the field of SSc and IIM where there is a huge number of misdiagnosed patients. The “seronegative gap” is one of the goals to address in the diagnosis of autoimmune diseases with the new methods to measure autoantibodies.

**Figure 5:**
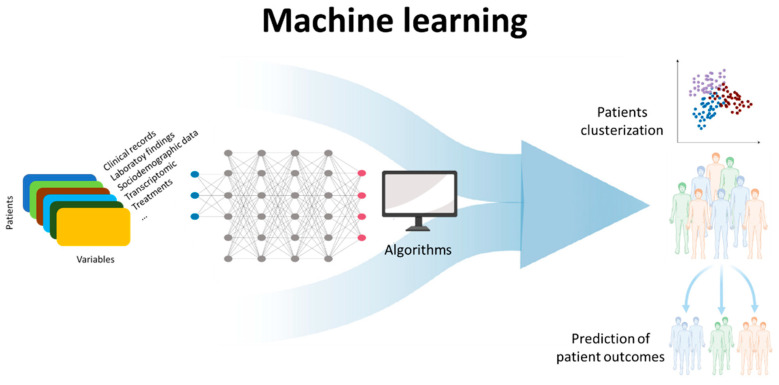
Schematic representation of a machine-learning process.

Another area of great interest but still to be exploited is the use of machine learning and applied artificial intelligence in autoimmune diseases and in ANA-associated SARDs. As autoimmune diseases are chronic, multifactorial conditions, through machine learning, a branch of the wider field of artificial intelligence, it is possible to extract patterns within patient data, and exploit these patterns to predict patient outcomes for improved clinical management [70,71] (Figure 5). However, these applications are not currently available, but are under development and present some technical limitations and other limitations including governance, reproducibility, and interpretation. Overcoming these limitations will enable machine learning methods to be more powerful in discovery and reducing ambiguity within translational medicine, allowing data-informed decision-making to deliver the next generation of diagnostics and therapeutics to patients quicker, at lowered costs, and at scale [72].

## 5. Discussion

**Figure 6:**
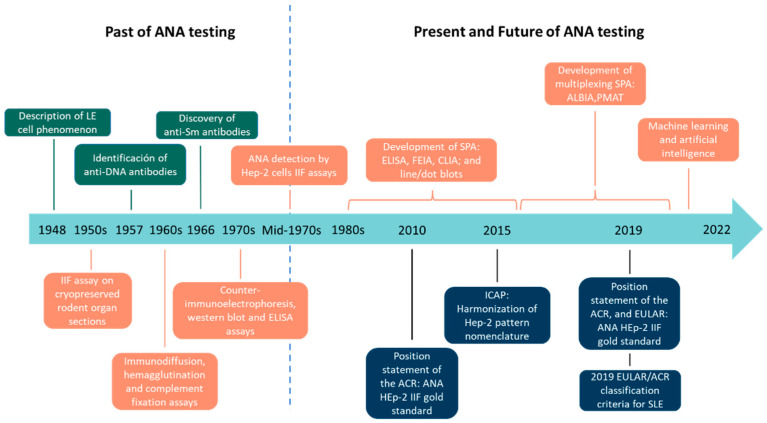
Chronological flow chart including the main events in the past, present, and future of antinuclear antibody (ANA) testing.

The existence of different techniques that allow us to evaluate the presence of the same parameter, ANAs in this case, should not be used as a limitation but as an advantage, since a test result with a high sensitivity, such as when using a HEp-2 IIF with a 1:80 cut-off titer, is useful for excluding the presence of disease, whereas a test result with a high specificity, for example, when using a SPA or HEp-2 IIF with a high cut-off value, is useful for confirming the presence of the disease. Therefore, we must be aware of the technique that is being used in order to correctly evaluate the obtained results. The performance of HEp-2 IIF and SPAs is disease-dependent. No single technique can correctly identify all patients with ANA-associated SARDs, so it is essential to combine the results obtained by various techniques to provide the most clinically valuable information. The identification of new biomarkers together with multianalyte platforms will help close the “seronegative gap” and correctly classify and diagnose patients with SARDs. Finally, artificial intelligence and machine learning are areas still to be exploited that, in the not-too-distant future, will help to extract patterns within patient data, and exploit these patterns to predict patient outcomes for improved clinical management. All the important advances mentioned throughout the present work, from the discovery of the LE cell phenomenon, through IIF using HEp-2 cells and SPAs, to multiplexed technologies and artificial intelligence, are summarized chronologically in Figure 6.

## Data Availability

Not applicable.
